# Supplementary material for: Are carbon emissions trading and green financial instruments synergistic? -Comprehensive quantitative research based on content analysis
Source: PLoS One. 2024 Mar 7;19(3):e0298601. doi: 10.1371/journal.pone.0298601 (PMC10919601; doi:10.1371/journal.pone.0298601)
Supplement: S1 Data — (ZIP) [file pone.0298601.s001.zip › Data/地级市Z_F工作报告.zip!/╣π╬≈/╣≡┴╓╩╨/└┤╫╘╣1⁄2╓┌║┼í╛┬φ┐╦╩2╛▌═°í┐_2010.docx]

各位代表：

现在，我代表市人民政府向大会作工作报告，请予审议，并请各位政协委员和其他列席人员提出意见。

一、2009年工作回顾

2009年是进入新世纪以来我市经济社会发展最为困难的一年，也是我们逆势奋进、经受严峻考验的一年，更是抢抓新机遇、实现新突破、取得新发展的一年。面对国际金融危机带来的严峻挑战，面对我市年初部分企业减产停工、农民工大量返乡、外贸萎缩、财政减收、经济增速下滑带来的巨大压力，我们认真贯彻落实中央和自治区的决策部署，在市委的正确领导下，深入学习实践科学发展观，坚持农业稳市、文化立市、旅游兴市、工业强市，紧紧围绕“保增长、保民生、保稳定、保持良好发展势头”的目标任务，切实采取非常办法、非常措施、非常力度、非常政策，扎实开展“项目建设年”、“服务企业年”、“党组织服务年”、“城市建设高潮年”活动，全市经济发展保持良好势头，社会事业全面进步，人民生活明显改善，较好地完成了市三届人大五次会议确定的各项目标任务。初步测算，实现地区生产总值940.55亿元，增长14.0％；财政收入97.64亿元，增长14.1%，其中一般预算收入55.15亿元，增长22.1%；全社会固定资产投资659.35亿元，增长35.7%；社会消费品零售总额330.92亿元，增长19.1%；节能减排达到自治区控制指标要求；城镇居民人均可支配收入16173元、农民人均纯收入4849元，实际分别增长11.4%和11.3%；城镇登记失业率控制在4.01%以内；人口自然增长率6.03‰；三次产业结构由上年的20.3︰42.8︰36.9[1]调整为18.9︰43.8︰37.3；全社会固定资产投资、城镇居民人均可支配收入提前两年完成“十一五”规划目标，财政收入、社会消费品零售总额、农民人均纯收入提前一年完成“十一五”规划目标。荣获全国社会治安综合治理最高奖“长安杯”、全国人口和计划生育综合改革示范市、全国科技进步先进市，连续四年获全国创建文明城市工作先进城市称号。

（一）全社会固定资产投资和项目建设实现重大突破

争取中央扩大内需投资项目1005个，新开工和在建投资1000万元以上建设项目1486项，全社会固定资产投资额超过“十五”时期的总和，比上年净增173.39亿元，是地市合并以来年均净增量的4倍，人均固定资产投资达1.29万元，是历年来我市重大项目建设数量最多、规模最大的一年。

全面落实重大项目责任制，强化项目集中联合审批，强力推进项目开工。全年上报建设用地2637.64公顷，获批2212.66公顷，均创历史最高水平。大力实施交通基础设施、城市建设、产业发展和民生工程等项目建设，燕京啤酒（桂林漓泉）股份有限公司年产20万吨啤酒生产线扩建、桂林福达集团汽车零部件产业园、桂林电力电容器有限责任公司特高压电力电容器生产基地、桂林尚科光伏技术有限责任公司太阳能CIGS电池及太阳能终端产品生产基地等产业项目竣工投产；两江国际机场A380备降场主体工程顺利完工。贵广铁路、湘桂铁路复线扩能改造、桂林市区至兴安高速公路、桂林南方水泥有限公司年产300万吨干法水泥（一期）、桂林恭城龙星锌业有限公司年产10万吨电锌技改扩建等续建重大项目加快推进。桂林市防洪及漓江补水枢纽工程——川江、小溶江水利枢纽工程等重大项目正式开工建设。阳朔至鹿寨高速公路、中国电力投资集团公司南方分公司资源金紫山风电场（一期）等重大项目正式启动。这些重大项目投资有力拉动了经济增长，为我市发展打下了坚实的基础。

（二）工业发展和园区建设实现重大突破

实现规模以上工业总产值726.46亿元，增长17.2%；全部工业增加值354.05亿元，增长16.7%，其中规模以上工业增加值244.65亿元，增长19.9％，利润总额32. 64亿元，增长28.3%。年销售收入超亿元企业由上年138户增加到151户。完成工业固定资产投资219.23亿元，增长31.5%；完成技术改造投资151.31亿元，增长135.3%，是“十五”时期的1.89倍，创历史最高水平。

以“五大五小”[2]产业为重点，建立健全企业发展服务机制，组建中小工业企业投资担保有限责任公司，切实从金融信贷、土地规划、技改贴息等方面帮助企业渡难关。“五大五小”产业总产值557.90亿元，占规模工业总产值的76.8%。加快工业园区（集中区）产业布局调整，加强园区基础设施建设，引导产业向园区集聚，工业园区新开工工业项目87个，完成投资58.20亿元，重点推进了中国化工橡胶桂林有限公司高等级子午线轮胎产业化、桂林电力电容器有限责任公司风力发电设备制造等一批资金投入大、科技含量高、带动能力强的工业项目，工业发展朝着规模化、集约化方向迈进。全市工业园区总产值突破300亿元，实现利税超35亿元；高新技术产业开发区技工贸总收入355亿元，园区已逐步成为推动工业增长的主要载体。

（三）城市建设和老城改造提升实现重大突破

以临桂新区开发建设和老城改造提升为重点，创新市县（区）城市建设联动机制，掀起了新一轮城市建设高潮。完成城镇固定资产投资545.20亿元，增长39.2%；城镇化率37.93%，比上年提高1.03个百分点。

完善了临桂新区中心区控制性详细规划、23个部门系统规划和项目专项规划。创业大厦、飞扬国际大厦、桂林大剧院、桂林博物馆、桂林图书馆、平桂西路等10多个重点项目启动建设。临桂新区市民广场等大型公共设施项目进入工程设计阶段；新征收土地3680亩，完成7个村庄安置点规划选址。老城改造提升重点实施了“三桥十路”[3]等市政交通基础设施建设，穿山桥、雉山桥、胜利桥、机场路城市段、六合路、中隐路二期、东二环路、西环路三期、黑山路、育才路等建成通车，漓江桥整修工程同步完成，城市交通路网结构改善，市区交通拥堵得到缓解。完成20公里城市道路710栋临街楼宇立面整治改造，主要道路和节点的绿化美化亮化工程同步推进，社区、庭院小游园及公共活动场所逐步完善。琴潭文化休闲娱乐园及一批特色街区建设加快，开展了一批城市道路桥梁等基础设施项目前期工作。深化拓展城乡清洁工程，开展市容专项整治行动，根据城市交通基础设施建设需要，搬迁及拆除违法建筑102万平方米，城市面貌进一步改善。荔浦滨江南岸新区、全州岳湾塘新区、龙胜桑江北区、资源城北新区、阳朔县城新区、平乐同乐新区、灌阳江东新区、灵川甘棠江城乡一体化建设核心区、恭城城乡一体化试点及永福、临桂、兴安县城靓化工程加速推进。县城路网、供水系统不断完善，重点镇建设加快进行，村容村貌整治得到加强。

（四）旅游发展和扩大消费实现重大突破

接待游客1860.08万人次，增长14.3%，其中入境游客129.03万人次；旅游总收入126.92亿元，增长26.6%，旅游业实现逆势上扬。第三产业增加值351.21亿元，增长14.1%。桂林成为国务院批准的全国首个以城市为单位建设国家旅游综合改革试验区的城市。

成功举办桂林国际山水文化旅游节、第三届联合国世界旅游组织旅游趋势与展望国际论坛，实现论坛永久落户桂林。召开旅游产业发展大会，扎实开展“旅游精品建设年”和“千车万人游桂林”活动，积极推进与广东、湖南等区域旅游合作。支持举办首届中国桂林创新创意文化节暨桂林动漫节、永福养生旅游福寿节、恭城桃花节、兴安桂林米粉节、阳朔渔火节、资源河灯歌节、临桂名人文化节、灌阳农具节等特色节庆活动，旅游业活力不断增强。认真贯彻落实中央、自治区一系列扩大内需政策措施，积极发展金融、物流、会展等服务业，切实做好农机下乡、汽车家电下乡、汽车家电以旧换新和“万村千乡”市场工程[4]，新建和改造农家店360家，家电下乡产品销售量达16.50万台，销售额2.95亿元，县以下消费品零售总额增长20.3%。商品房成交面积124.96万平方米，增长57.6％。

（五）县域经济和特色农业实现重大突破

12县完成地区生产总值646.63亿元，增长14.9%，占全市的68.8%；完成全社会固定资产投资513.54亿元，增长33.9%，占全市的77.9%；完成规模以上工业总产值448.40亿元，增长23.1%，占全市的61.7%；财政收入42.94亿元，增长13.5%，县域经济综合实力显著增强，成为推动经济发展的重要力量。

大力推进漓江、湘江、资江三大流域特色农业示范区建设，农业规模化、标准化、品牌化、产业化建设成绩突出。第一产业增加值177.34亿元，增长5.3%。粮食总产量193.30万吨，连续六年实现增产。水果、蔬菜、食用菌种植面积和总产量持续增加，位居全区第一。家禽生产首次突破亿羽大关。资源县入选中国果菜无公害十强县。永福县“永福香”牌香米获全区粮食行业唯一著名商标。新增农产品标准化生产基地1.58万亩、万头标准化养殖场15个。农民专业合作社1315家，通过订单合同带动农户11万户。落实国家惠农政策补贴资金14.10亿元，比上年增加8.30亿元。集体林权改革试点工作积极推进，完成集体林地勘界确权面积522.97万亩，占自治区下达任务的140.8%。第二批82个贫困村“整村推进”全部通过自治区验收，优秀率达100%，减少贫困人口4万人。开展农业技术培训112.47万人次，农村劳动力转移培训13.87万人次。

（六）财政和融资工作实现重大突破

全年争取中央和自治区扩大内需投资13.21亿元，争取地方政府债券额度6亿元，新增到位市本级各投融资公司银行贷款资金98.88亿元，是历年来我市争取资金最多的一年。全市金融机构贷款余额达657.14亿元，其中新增贷款210.11亿元，增长47.0%，新增贷款总量和增幅均创历史新高。

不断深化财政体制机制改革，完善公共财政体系，调整优化支出结构，严格控制一般性支出，公务车辆购置和运行费、出国经费和公务接待费均压缩10%以上，切实保障重点项目建设及“三农”、义务教育、医药卫生、民生等公共支出。出台鼓励金融机构支持地方经济发展政策措施，财政与金融的联动机制进一步完善。与国家开发银行签订73.90亿元银团贷款合同，浦发银行桂林支行正式开业，兴业银行、光大银行等金融机构已获准筹建桂林分支机构。积极推进政府投融资公司与中介机构签订10亿元企业债券合作协议。促成桂林三金药业股份有限公司成为国家重启新股发行后首家上市公司，成功募集资金9.10亿元。我市上市企业数量和募集资金居全区前列。

（七）开放合作和改革创新取得明显成效

全市新签引进市外境内项目537个，总投资327.84亿元，增长25.2％。实施市外境内项目961个，实际到位资金280.94亿元。新批外商投资企业20家，实际利用外资2.02亿美元，位居全区第二。

主动融入北部湾、湘桂、桂台等多区域合作，积极参加第六届中国—东盟博览会、第五届泛珠三角区域合作经贸洽谈会、第五届桂台经贸交流会、第十二届中国（重庆）投资洽谈暨全球采购会。精心组织与日本熊本市结为友好城市30周年纪念活动，大力推进与国内外城市交流合作。实现了桂林与台湾双向直航。组织招商队伍赴港澳台、京沪浙闽粤等发达地区开展产业招商、专题招商、以商招商活动，成功引进美国沃尔玛百货有限公司、中国有色金属总公司、中国电力投资集团公司等国际国内知名企业和中央大企业，积极促成中国化工集团公司、广州白云电器集团有限公司、上海铭源实业集团有限公司、上海复星药业（集团）股份有限公司等企业在我市新上项目、扩大投资。国有企业改革和重组取得新成绩，引入市外到位资金4.30亿元。启动兴安县新型农村养老保险制度改革试点，政府机构、医药卫生、社会保障、社会事业等体制改革取得实质性进展。

　　（八）生态建设和节能减排取得明显成效

以建设生态山水名城为目标，强力推行环境保护目标责任制，加强城市环境综合整治和节能减排。《桂林生态市建设规划》、12县生态县建设规划全部通过专家论证。成立漓江流域管理委员会，研究制定科学保护漓江工作规划，打击漓江非法竹筏营运和整顿漓江鱼餐馆成效明显。渔政执法被评为全国文明执法窗口单位。“两江四湖”二期工程——桃花江、南溪河、小东江综合整治工程，山口垃圾填埋场、临桂新区及东区污水处理厂工程启动建设。12县污水处理项目全部开工，临桂、灵川、兴安、荔浦、雁山污水处理系统工程及上窑污水处理厂改造工程竣工使用。城镇（包括12县）污水处理率达55.0%。加快淘汰不符合国家产业政策的小砖瓦窑、小水泥、小造纸等落后生产能力。大力推广工业、建筑和公共事业节能新技术、新材料、新设备。全市万元生产总值能耗降低4.33％，45家重点耗能企业节能12万吨标准煤，化学需氧量、二氧化硫排放总量分别控制在4.48万吨和6.46万吨以内。植树造林41.10万亩、毛竹造林8.42万亩，森林覆盖率达66.5%。累计建沼气池55.77万座，可建池入户率达76.7%，继续保持全国领先水平。完成全国第二次土地调查各项工作。“国家园林城市”顺利通过复查，环境保护“城考”继续保持全区第一。

（九）保障和改善民生取得明显成效

建立健全政策扶持、创业培训、创业服务工作机制，帮助困难企业减负8365.63万元，新增城镇就业7.23万人、下岗失业人员再就业1.78万人，返乡农民工就业22.58万人。全面实施城镇居民基本医疗保险制度和城市居民医疗救助制度，全市基本养老、失业、医疗等社会保险参保人数分别达35.79万、25.86万、100.36万。解决10859名市属国有关闭破产和困难企业退休人员医疗保险问题。市区企业退休人员养老金人均月增资133元。5城区城市居民低保标准提高至每人每月260元、12县城镇居民低保标准提高至每人每月200元以上，农村低保标准提高至每人每年1200元以上，累计发放低保金2.79亿元，实现40.11万低保对象应保尽保。投资950万元，新建19个乡镇敬老院。住房公积金制度覆盖面进一步扩大，社会福利逐步向适度普惠型发展，建成经济适用房26.5万平方米。

全年投入16.02亿元，全面完成10项惠民工程。城镇居民基本医疗保险参保人数达47.28万，完成年度计划的236.4%；新型农村合作医疗参加人数达359.39万，参合率93.3％。完成20所中等职业学校改（扩）建工程年度投资任务；资助23037名普通高中贫困生和贫困大学新生上学，发放资金1386.72万元。完成3000个自然村（屯）新农村建设规划编制、55个自然村新农村建设试点。实施1500个自然村（屯）内道路硬化，完成30个行政村通油路或水泥路项目。完成1083户农村贫困残疾人危房改造。修建农村饮水安全工程359处，解决20.03万农村人口饮水安全问题。新建农村沼气池2.45万座。对167个50户以上连片木结构民族村寨实施防火综合整治。新建31个计生服务站（所）、28个司法所。新增1897个20户以上自然村通广播电视。完成3.80万平方米廉租房建设任务。訾洲生态公园建设基本完成。西环路、七星路、龙隐路、滨江路、五美路路灯改造工程顺利完工。市区重要交通节点改造治理取得显著成效。

（十）社会事业和社会管理取得明显成效

全社会科技投入14.20亿元，实施技术创新项目532项，推广科技成果323项；国家知识产权示范城市创建工作扎实推进，专利申请量和授权量位居全区前列；12县5城区全部通过2007—2008年全国科技进步考核；荔浦县跻身首批国家知识产权强县。新建改建校舍13.10万平方米，发放中等职业学校国家助学金1787.87万元；教育质量不断提高，高考成绩名列全区前列，中职教育技能比赛成绩全区第一。新建500家农家书屋、21个乡镇文化站；文化信息资源共享工程[5]进展顺利，7个县级支中心、35个乡镇基层服务点、95个村级基层服务点投入使用；桂林百姓大舞台成功举办25场，百姓大舞台、百姓文化大讲坛和市民读书月活动影响日益扩大，受众达百万人次；漓江之声被评为全国首批群众文化品牌，桂剧《灵渠长歌》获第七届广西戏剧展演桂花金奖。《桂林年鉴》获第四届全国年鉴编纂出版质量综合评比一等奖。新建扩建城乡医疗卫生用房10万平方米，第二轮农村初级卫生保健工作提前一年达标；创建文明卫生村427个，完成农村改厕1.05万座；甲型H1N1流感疫情防控取得阶段性成效；荣获无偿献血全国先进城市。全民健身蓬勃发展，竞技体育成绩斐然，新建农民健身工程208个，桂林籍运动员取得世界青年举重锦标赛1金1银、第11届全运会１金２银１铜的优异成绩。人口和计生工作整体水平继续保持全区领先地位，农村“诚信计生”工作得到国家计生委高度评价，阳朔县、七星区获全国计划生育优质服务县（区）。恭城瑶族自治县被国务院评为全国民族团结进步模范集体。

社会治安防控体系更加完善，社会治安综合治理更加有效，圆满完成庆祝新中国成立60周年等重大活动安全保卫任务。“五五”普法教育、法治城市和法治县区创建活动扎实开展。认真开展矛盾纠纷排查化解、领导干部“大接访”及机关干部带案下访活动，调解化解各类矛盾纠纷2484件，审结行政复议案件194件。应急体系不断完善，突发性事件得到及时有效处置，产品质量和食品药品安全监管力度不断加大。全面开展安全生产“三项行动”和“三项建设”[6]，安全生产形势总体保持稳定。国防动员、民兵预备役力量建设、人民防空及双拥工作得到加强。宗教、人事、统计、新闻出版、编制、接待、机关事务、档案、供销、防震减灾、侨务、发展研究、社会科学、妇女儿童、老龄等事业取得新进展，中直、区直驻桂单位不断取得新成绩。

一年来，我们坚持科学决策、民主决策，转变政府职能，破解制约科学发展的突出问题，提高政府执行力、控制力和公信力。坚持重大事项向市人大及其常委会报告和市政协通报制度，办理市人大代表建议、批评和意见69件，市政协提案298件，办理质量得到提高，办结率达100%。坚持广泛听取各民主党派、工商联、无党派人士意见，支持工会、共青团、妇联等人民团体更好发挥作用，密切与人民群众沟通联系。坚持完善行政监察和审计监督，切实从源头上预防和治理腐败。

在充分肯定成绩的同时，我们也清醒地看到，当前国际金融危机仍未结束，我市加快发展还面临诸多困难和问题，主要表现在：受国际金融危机冲击，部分重点行业、企业生产经营困难，效益下滑，大宗商品进出口大幅下降的状况还没有根本好转；农民持续增收困难，就业和社会保障压力不断增大；重大项目储备不足，部分重点项目进展缓慢；县区发展不够平衡；部分干部作风亟待改进，发展软环境需进一步优化，城市管理水平有待提高。我们一定高度重视，积极应对，千方百计克服存在的困难和问题，促进经济社会又好又快发展。

回顾一年来的政府工作，我们沉着应对国际金融危机的严重冲击，保持了经济社会平稳较快发展，创造了战胜危机、走出困境、逆势而上的显著成绩，积累了十分宝贵的经验：一是必须全面贯彻落实胡锦涛总书记“四保”重要指示精神和中央、自治区一系列决策部署，团结拼搏，克难攻坚，创造性地开展工作。二是必须采取非常办法、非常措施、非常力度、非常政策，以新思路谋求新发展，以新举措赢得新突破，推动各项事业加快发展。三是必须以全社会固定资产投资、技术改造投资、金融机构新增贷款三项工作为重要抓手，坚持交通优先、产业优先、园区优先，通过大项目、大投入，实现大发展。四是必须善于从不断变化的复杂形势中敏锐地发现和把握机遇，充分利用各种资源，创新投融资平台，多渠道筹措项目建设资金，为加速经济发展提供重要支撑。五是必须在市委的正确领导和市人大、市政协的监督下，转变政府职能，转变工作作风，加强监督检查，切实把着力点放在抓落实上，提高行政效率，充分激发全社会的创造活力。

各位代表，过去一年的成绩来之极为不易，凝聚了全市人民的智慧和汗水，是全市上下齐心协力、扎实工作的结果。在此，我谨代表市人民政府，向全市各族人民，向给予政府工作支持和监督的人大代表、政协委员，向各民主党派、工商联、各人民团体和社会各界人士，向驻桂各单位、驻桂人民解放军、武警官兵，以及所有关心支持我市发展的朋友们，表示衷心的感谢！

二、2010年工作总体要求和目标任务

今年是经济社会发展形势较为复杂的一年，是实施“十一五”规划和科学发展三年计划的最后一年。做好今年的工作，保持经济平稳较快发展，为“十二五”规划实施打下坚实基础，具有十分重要的意义。

当前，我国经济形势总体回升向好，中央继续实施积极的财政政策和适度宽松的货币政策，实施新一轮西部大开发战略，尤其是国务院《关于进一步促进广西经济社会发展的若干意见》的出台，推动我市加快发展的政策效应将进一步凸显。中国—东盟自由贸易区建成，我们与东盟的贸易与投资将进一步扩大。广西已进入经济社会快速协调持续发展和全方位开放的新阶段，自治区党委、政府高度重视我市的发展，为我们争取政策、资金和项目提供了难得的机遇。但是，我们也应当看到我市经济回升向好的基础还不稳固，不确定不稳定的因素难以预料，制约经济社会发展的长期性和深层次问题还没有根本解决。我们必须保持清醒头脑，把应对各种挑战的措施准备得更周全一些，把各项工作做得更扎实一些。我们必须坚持发展第一要务不动摇，把加快发展作为最大的政治、最硬的道理、最紧迫的任务，一心一意谋发展，聚精会神搞建设，全心全意惠民生，尽心尽力促和谐，绝不辜负全市人民的期望和重托。

今年政府工作总体要求是：以邓小平理论和“三个代表”重要思想为指导，深入贯彻落实科学发展观，认真落实中央、自治区的决策部署和国务院《关于进一步促进广西经济社会发展的若干意见》以及市委三届九次全会精神，紧紧抓住建设桂林国家旅游综合改革试验区的历史机遇，着力开展以“城市建设、交通基础设施建设、园区建设、城乡风貌建设”为重点的项目建设大会战，切实增强经济社会发展后劲；着力推动经济发展方式转变和经济结构调整，全面提高经济质量和效益；着力加快工业、农业、旅游业、现代服务业发展，进一步做大做强支柱产业；更加注重统筹各项社会事业全面发展，更加注重改善民生，更加注重生态环境建设，更加注重推进改革和自主创新，增强经济增长活力和动力，为保持和扩大经济社会发展良好势头、加快建设富裕文明和谐新桂林而努力奋斗。

主要经济社会发展预期目标是：地区生产总值增长12%，全社会固定资产投资增长30.5%，财政收入增长13%，社会消费品零售总额增长16%，万元生产总值能耗降低4.21%，二氧化硫、化学需氧量排放总量控制在自治区下达任务指标以内，主要江河3类水质达标率100%，城镇新增就业5.6万人，城镇登记失业率控制在4.5%以内，城镇居民人均可支配收入增长10%，农民人均纯收入增长10%，人口自然增长率控制在7‰以内。

做好今年政府工作，要切实把握好以下原则：一是坚持把转变经济发展方式作为深入贯彻落实科学发展观的重要目标和战略举措。二是坚持把保持经济平稳较快发展作为首要任务。三是坚持把扩大内需作为促进经济增长的主要着力点。四是坚持把推进结构调整和自主创新作为转变发展方式的主攻方向。五是坚持把城市建设、交通基础设施建设、园区建设、城乡风貌建设作为加快发展的龙头和工作重点。六是坚持把加快城镇化作为统筹城乡协调发展的切入点。七是坚持把改革创新和扩大开放合作作为推进科学发展的根本动力和强大活力。八是坚持把保障和改善民生作为政府工作的出发点和落脚点。

三、2010年主要工作部署

实现今年经济社会发展目标，必须继续采取非常办法、非常措施、非常力度、非常政策，继续扎实开展“项目建设年”、“服务企业年”、“城市建设高潮年”活动，着重抓好以下九方面工作：

（一）狠抓项目投资，强劲拉动经济实现新增长

继续把抓投入上项目作为全年工作的重中之重，扎实开展以城市建设、交通基础设施建设、园区建设、城乡风貌建设为重点的项目建设大会战。全年完成全社会固定资产投资860亿元，其中城镇固定资产投资705亿元。统筹推进重点项目239项，年度投资236亿元。

强力推进“四大建设”。城市建设力争完成投资200亿元，突出抓好以临桂新区和老城改造“1212”[7]工程为重点的城市基础设施和公共服务设施建设。交通水利基础设施建设力争完成投资120亿元，积极推进以“两铁五高三库”[8]为代表的重点项目建设，争取湖南新宁经资源至兴安高速公路、灌阳经恭城至平乐高速公路尽快立项，加快临苏公路建设以及国道、省道、县道改造，深入实施村村通工程；推进两江国际机场航站楼扩建工程建设。工业园区建设力争完成投资80亿元，大力推进高新技术产业开发区、苏桥经济开发区、西城经济开发区建设，全力打造百亿元工业园区。城乡风貌和社会事业项目建设力争完成投资43亿元，以桂柳、桂梧、桂黄高速公路及桂阳公路沿线、漓江两岸和12县城为重点，全面加快城乡风貌改造，带动重点镇建设和新农村建设。

强化项目管理和前期工作。继续按照“一个建设项目、一名主管领导、一个工作机构、一个计划目标、一抓到底”的工作机制，建立健全项目建设责任制，加大项目前期经费投入，完善部门联合审批制度，推进并联式审批工作，强化项目督查协调和工程质量监理，着力解决项目用地、环评、资金、搬迁、安置等突出问题，千方百计促进新建项目集中开工，千方百计推进续建项目早日竣工达产。着力策划一批有利于长远发展的优势产业、基础设施和社会事业项目，形成规划储备一批、开工建设一批、建成投产一批重大项目工作格局。

加强财政和金融工作。加强财源建设和收入征管，不断壮大财政实力。继续深化各项财政改革，加强政府投资项目资金的调度与监管，做好项目资金保障工作。创新投融资模式，充分发挥投融资平台的作用，增强财政资金对民间投资的拉动力；把握中央、自治区投资走向，加强项目编制和申报，积极争取中央、自治区更多资金支持。完善政银企合作机制，大力推进“引银入桂”工作，积极促成兴业银行桂林分行和光大银行桂林支行尽早开业。鼓励和支持市商业银行立足桂林、面向广西，做大做强。充分发挥工业担保公司和中小企业服务中心平台作用，积极推进中小企业服务体系建设，切实加强对中小企业的扶持力度。鼓励发展法律服务、资产评估、融资担保、信用评估等中介组织，支持企业申请发行企业债券。力争1家企业上市融资。

（二）转变发展方式，推进以高新技术产业为先导的现代工业实现新发展

坚持以高新技术产业为先导，充分利用国家给予我市发展工业的有关政策，以“五大五小”优势产业为支柱，以强优企业为龙头，以园区为载体，继续加大投入，加快用高新技术改造提升传统产业，全力推进产业结构转型升级。工业总产值突破1200亿元，增长18%以上；工业增加值420亿元，增长16%以上；技术改造投资确保增长35.5%。

加快高新技术产业发展。进一步办好桂林国家高新技术产业开发区，充分发挥高新区的引领、示范、辐射、带动作用，促进产业结构优化升级，实现高新技术产业跨越式发展。全面推进桂磨高新技术产业带建设，做大做强做优电子信息、生物制药、新材料、新能源等高技术产业；大力发展软件服务外包、动漫等新兴产业。重点推进桂林星辰电力电子有限公司、中国电子科技集团公司桂林激光通信研究所、中国机械工业集团公司桂林电器科学研究所、桂林南药股份有限公司、桂林海威科技有限公司、桂林啄木鸟医疗器械有限公司等科技型企业发展壮大。

　　加快优势产业发展。在继续推进“五大五小”产业发展的基础上，加快推进机械、汽配、橡胶、医药、特色农林产品精深加工等产业升级换代。机械产业重点发展电工电器、工程机械、橡胶机械、机床工具、仪器仪表和机电一体化。汽配产业重点发展油电混合动力、汽车电子、汽车底盘及新能源客车。橡胶产业重点发展高等级子午线轮胎、乳胶及橡胶制品。医药产业重点发展现代中药、生物制药、化学药及医疗器械。特色农林产品精深加工重点发展粮油、果蔬、畜禽、竹木等产品加工。鼓励建设中间产品、元器件、零部件、配件等生产资料市场，支持建立行业协会等社会组织，努力形成区域化布局、专业化分工、配套化生产的特色产业集群。

加快企业结构调整。坚持以技术改造提升优势产业，坚持以大企业大集团带动优势产业，继续抓好招大引强、培优扶强工作，促进信息化与工业化融合，着力在科技攻关、技术改造、煤电油运、市场营销、融资担保等方面提供服务。计划实施单项投资500万元以上工业项目382项，总投资495.35亿元。其中，新开工项目143项，总投资83.64亿元，年度投资58.68亿元，重点推进桂林大宇客车有限公司客车系列产品产业化及整厂搬迁改造、桂林娃哈哈食品有限公司饮料生产线扩建三期工程、桂林立白日化有限公司新型洗衣粉和洗洁精扩能更新改造、桂林尚科光伏技术有限公司CIGS太阳能电池二期等项目开工建设。续建项目226项，总投资391.45亿元，年度投资164.77亿元，重点加快中国化工橡胶桂林有限公司高等级子午线轮胎产业化、桂林客车发展有限公司年产12000辆轻型客车搬迁改造、桂林客车工业集团有限公司年产8000辆客车及底盘生产线搬迁改造、桂林电力电容器有限公司风能发电设备制造、桂林康密劳铁合金有限公司锰系列合金生产搬迁及产品升级、桂林福达集团年产10万吨精密锻造件锻造中心、桂林三金药业股份有限公司现代中药产业化、中国电力投资集团公司南方分公司资源金紫山风电场和桂林电网改造、桂林天和药业股份有限公司年产6亿片贴膏产业化等建设项目进程。

加快重点工业园区建设。进一步完善园区管理体制，按照布局集中、用地集约、产业集聚的要求，加强工业园区建设及产业规划，做好与城镇新一轮土地利用规划修编的衔接，加大工业土地储备力度，拓展园区发展空间。继续开展以“路、水、电、网、房、校、所”[9]为重点的园区基础设施建设大会战，高层次大规模承接产业转移，促进工业项目向园区集聚，加快发展产业互补、功能齐全、特色鲜明的园区经济。苏桥经济开发区重点抓好橡胶产业基地、客车生产基地建设，加快汽车及零部件企业聚集，支持国电永福发电有限公司上大压小热电联产项目建设。西城经济开发区重点加快机械及汽车零部件、现代中药、食品饮料等产业建设，着力打造临苏工业长廊。各县工业集中区要以特色农产品加工和优势资源型工业为重点，加快发展与大型企业集团相配套的产业集群，大力推动中小企业与大型企业协作，着力打造一批工业强县。进一步加大政策支持力度，积极推进工业企业“退二进三”，力争新入园企业和项目100个以上，园区规模以上工业增加值增长25%以上。

（三）优化农业结构，促进以特色农业为主导的现代农业实现新进步

按照“稳粮保供给，增收惠民生，改革促统筹，强基增后劲”的要求，以结构调整为重点，以规模农业、品牌农业、生态农业、观光农业为核心，全面提升特色效益农业发展水平，促进农业增产，农民增收。第一产业增加值增长5%以上；力争农民人均纯收入达到全国平均水平。

　　加快发展现代农业。深入实施漓江、湘江、资江和桂江流域及山区现代农业示范区建设，着力提高特色效益农业规模化、标准化、品牌化、产业化水平。大力推广超级稻、测土配方施肥和农作物间套种等新品种、新技术，着力建设优质粮食、果蔬、畜禽、中药材、竹木花卉等一批优势农产品生产基地。抓好粮源基地建设，提高粮食综合生产能力。抓好果蔬标准园建设，提升出口果蔬品质。加快特色种养业发展，壮大食用菌产业，鼓励发展特色养殖。积极推进竹木加工贸易、人造板、竹制品、木衣架等产业发展。扶持发展农业龙头企业和农民专业合作社，加快农业产业化进程，确保食品加工业和林产工业产值分别突破150亿元、55亿元。积极创建全国循环农业示范市，加快现代设施农业、生态循环农业和旅游观光农业发展，推进“魅力桂林乡村农业四季游”20条观光农业线路建设，不断提高农业特色节庆活动水平，加快休闲农业发展。

　　完善农业服务体系。建立健全农业社会化服务体系，提高农村公共服务能力。加快基层农业技术推广体系改革与建设，着力构建主导品种和主推技术进村入户、下地下田的农业科技推广新机制，提高科技兴农水平。加强动植物保护，着力完善动植物疫病预警与控制体系，落实重大动植物疫病防控责任制，提高农业抗风险能力。加强农产品检测体系建设，着力健全农产品质量可追溯制度，确保农产品各环节质量安全。创新农业金融服务体系，加快发展农户小额信贷。规范农村经营市场，全面改善农村经营环境。

加强农村基础建设。全面落实各项强农惠农政策。引导和扶持农村土地规范流转，全力抓好集体林权制度改革，完成1600万亩集体林权主体改革任务，积极推进配套改革。积极推动城市基础设施和公共服务向农村延伸，推进城乡结合部和景区周边农村率先一体化发展。组织150个自然村开展“普惠制”新农村建设试点，继续创建400个文明卫生村，建设农村卫生厕所1.55万座。加强农田水利建设，启动89座病险水库除险加固，提高防汛抗旱和防灾减灾能力。实施水库移民新村建设工程。加大扶贫开发力度，高标准完成第三批82个贫困村“整村推进”任务。继续实施“千乡万村现代农机装备推进工程”，提高农业综合机械化水平。实施农技培训工程和农民转移就业“阳光工程”，大力促进农民就业创业和农村富余劳动力转移。

（四）提升壮大旅游业，推动以旅游为龙头的现代服务业实现新进展

充分把握建设桂林国家旅游综合改革试验区的重大历史机遇，勇于改革，敢为人先，统筹规划，大胆创新，积极争取国家、各部委和自治区的支持，全力推进建设桂林国家旅游综合改革试验区的各项工作，加快旅游集散地建设，着力打造国际旅游胜地和大桂林国际旅游目的地，把旅游业培育成战略性支柱产业和人民群众更加满意的现代服务业，全面提升以旅游为龙头的现代服务业发展质量和效益。第三产业增加值增长11%，社会消费品零售总额增长16%，接待国内外游客增长5%，旅游总收入增长10%。

加快旅游业转型升级。全面启动旅游“二次创业”，整合旅游资源，丰富和完善以市区为中心、各县为节点的大桂林旅游圈，发展一批旅游强县和特色旅游小城镇，建设多样化、特色化、品牌化的旅游产品体系。推出历史文化旅游精品线路，推进漓江流域特色旅游富民项目建设，拓展观光旅游。实施“精品景区工程”和“精品酒店工程”，建设一批特色旅游文化街区和精品旅游设施，发展高端旅游。打造“两江四湖”滨水旅游休闲带、环桂林老城休闲度假带、阳朔“十里画廊”等生态休闲旅游区，发展休闲旅游。全力办好第四届联合国世界旅游组织旅游趋势与展望国际论坛和首届中国桂林国际旅游博览会，完善会展会议配套功能，发展会展商务旅游。加快以旅游交通、旅游服务、旅游安全为重点的基础设施建设，搭建旅游公共服务平台。充分发挥桂林旅游高等专科学校广西东盟旅游人才教育培训基地的作用，积极培养旅游人才。加强旅游宣传营销，强化区域合作，全方位拓展客源市场。积极引进国内外有实力的大型旅游企业，做大做强一批旅游骨干企业和知名企业。进一步加强旅游管理，依法规范和整治旅游市场，营造舒适和谐的旅游环境。

大力发展现代服务业。编制临桂新区和老城区商业网点规划，出台加快发展商贸业政策措施，加快发展信息、物流、金融、咨询、法律等服务业。大力发展新型流通业态，积极引进国内外知名大型商贸企业和品牌，培育2—3家销售收入超10亿元的大型商贸流通企业。推进桂林市现代物流园区、现代物流配送中心前期工作。大力发展休闲消费性服务业，建设一批时尚动感、饮食休闲、旅游娱乐等特色街区，进一步发展特色餐饮、风味小吃，创建有特色、高品质的饮食品牌。合理布局，发展壮大旅游商品、农产品、建材、五金机电、汽车及汽车配件等商贸批发市场、专业市场和大型商业零售网点，构建新兴特色商圈。

积极扩大消费需求。大力培育文化娱乐、教育培训、体育保健、家政服务等消费热点，促进居民消费。继续实施扩大城乡居民消费“510”活动[10]和家电下乡工作，落实农机购置补贴，大力推进农家店、农资配送中心、“双百市场”[11]、标准化农产品批发市场、农贸市场改造升级工程等农村物流服务体系和“八桂放心肉”工程建设，促进农村消费。建设经济适用房25万平方米，着力解决低收入家庭住房困难；增加普通商品住房供给，支持居民自住和改善性购房需求；积极推动旅游房产、商务房产，促进房地产业平稳健康发展。扩大和完善消费信贷，加大市场监管和商务执法力度，努力营造安全、放心、便利的消费环境，引导消费结构升级。

（五）加快城镇化进程，继续掀起城市建设新高潮

坚持以临桂新区建设和老城改造提升为重点，完成《桂林市城市总体规划》、《桂林市土地利用总体规划》修编及上报，完善城市功能，改善人居环境，着力提高城乡规划、建设和管理水平。

全面推进新区建设。坚定不移地实施“保护漓江，发展临桂，再造一个新桂林”发展战略，着力破解土地征用、搬迁安置、投资融资、体制机制等制约新区建设的瓶颈问题，举全市之力，加快新区建设，切实把新区建设作为城市建设的主战场，迅速构建新区城市框架。继续统筹推进新区规划，完善城市水系、路网等规划布局。千方百计加大投资力度，新开工项目65项，总投资199亿元，确保完成年度投资25亿元。强力推进创业大厦、桂林大剧院、桂林博物馆、桂林图书馆、山水大道、新中路、桂林市职业教育中心学校临桂分校、桂林医学院临桂新校区、污水处理系统工程、万福路供水加压站等已开工项目建设进度，力争创业大厦主体工程年底基本完工。加快实施市民广场、中央公园、金融大厦、广电中心、报业大厦、水利防洪排涝、湖塘水系工程以及搬迁安置点等项目建设。完成新区中心区范围土地征用。积极推进金融、保险、商贸、物流、医疗、教育等优势资源向新区转移，大力引进有实力的企业投资新区开发建设，增强新区建设生机与活力。

提升完善老城功能。加快老城控制性详细规划编制，完善漓江小流域综合整治以及重要街区规划设计。按照完善功能、凸显特色、提升品位、优化环境的要求，实施市容环境提升行动计划[12]，全力抓好特色城市建设工程、城乡风貌改造工程、城市主次干道美化亮化工程、园林绿化建设工程、市容市貌管理工程。加快推进“两江四湖”二期工程，加快建设秀峰区琴潭文化休闲娱乐园、叠彩区商贸物流园、象山区旅游度假园、高新（七星）区产业园、雁山区科教园，抓好特色街区改造。完善城市路网结构，全面实施西二环路、万福东路、龙门大桥等一批路桥基础设施重大项目，建设总里程约94.6公里。编制城市交通专项规划，加强市内交通道路秩序整治，实施机场路收费站迁建项目，启动汽车总站分期转迁。实施“两江四湖”滨水街区城市立面改造，滨江旅游景点和城区重要节点改造，启动漓东公园、黑山植物园二期建设，抓好猫儿山公园、净瓶山公园项目征地搬迁工作。健全城市管理长效机制，加快数字化城市管理工程建设，做好高新（七星）区数字化城市试点工作。深化拓展城乡清洁工程，继续推进“竹筒房”、“城中村”改造。巩固提升创建国家卫生城工作成果，创建全国无障碍示范城市。

加强特色城镇建设。加快完善县城、小城镇总体规划和土地规划，强力推进县城基础设施建设，着力提升城镇综合承载能力和服务能力。继续推进龙胜、荔浦、全州、资源、阳朔、平乐、灌阳等县新区建设以及灵川甘棠江城乡一体化试验区和恭城城乡一体化建设。坚持特色立城，完成桂柳、桂梧、桂黄高速公路以及桂阳公路沿线500米可视范围内和漓江风景名胜区内漓江两岸城镇、村庄风貌改造，以及12县县城和一批特色乡镇立面改造。抓好兴安、平乐等县城防洪堤工程建设。全面启动12县农村危房改造。

　　（六）推动文化创新，培育文化桂林建设新亮点

坚持文化立市，把建设社会主义核心价值体系贯穿于文化建设全过程，着力培育城市人文精神，以文化创新促进文化大发展、大繁荣，全面提升文化创造力、凝聚力、辐射力。

制定文化发展规划。坚持一手抓文化事业繁荣，一手抓文化产业发展，加快编制《桂林市文化发展规划》，实施文化精神工程、文化景观工程、文化精品工程、文化惠民工程、文化管理工程，打造广西文化强市，不断增强文化软实力，满足人民群众日益增长的精神文化需求，为实现我市经济社会又好又快发展提供强大的精神动力和文化条件。

加强文化基础建设。全力推进“一院两馆”[13]重大项目建设，着力抓好甑皮岩遗址博物馆二期工程、以靖江王陵大遗址保护工程为核心的历史文化建设项目、八路军桂林办事处旧址红色旅游等项目实施。加强公共文化服务体系建设，积极推进社区文化中心、乡镇综合文化站、村级文化室、文化信息资源共享、农家书屋等工程，新建乡镇综合文化站32个、农家书屋369个。加强文物和非物质文化遗产保护，做好第三次全国文物普查工作，保护和建设具有桂林特色的文化名镇、名街、名村。

推进文化精品建设。继续打造百姓大舞台、百姓文化大讲坛、漓江之声、市民读书月、五通农民画等文化品牌，以品牌文化带动社区、企业、农村群众文化蓬勃发展。鼓励支持文化人才深入基层体验生活，创作一批具有桂林特色、时代风采和市场前景的舞台艺术精品，推出一批反映现实生活、体现时代精神的优秀文艺精品。举办第二届中国山水画艺术双年展，推进文化交流合作，扩大优秀特色文化产品的对外影响力和国际竞争力，打造山水实景演出国际品牌。完善人才培养、引进和竞争机制，着力培育引领各领域创新发展的文化人才。繁荣发展社会科学，完成《桂林市志》编纂出版。

发展文化创意产业。制定文化创意产业发展规划，大力推进高新区创意产业园、雁山动漫文化产业园等园区建设。重点培育发展演艺娱乐、动漫游戏、出版发行、印刷制作、广告会展、网络数字等产业，着力建设动漫基地。加强文化市场管理，积极培育文化艺术品交易市场和文化要素市场。充分发挥文化产业投资公司融资作用，鼓励民营资本进入文化产业领域。

（七）推进改革开放，争创活力桂林建设新优势

继续解放思想，扩大开放合作，着力推进关键环节的改革，消除体制机制障碍，激发全社会创造活力。

推进重点领域改革。全面推进医药卫生体制改革，探索公立医院改革，构建新型城市医疗卫生服务体系。进一步抓好新型农村合作医疗工作。推进兴安县新型农村养老保险制度改革试点，实现参保覆盖率80%以上。深化资源性产品价格和环保收费改革。推进国有、集体企业和供销体制改革，加强国有资产监管，促进国有资产保值增值。大力发展非公有制经济，支持非公有制企业进入基础设施、公用事业和社会事业等领域。

建设创新型桂林。深入开展全国科技进步示范市和国家知识产权示范城市创建活动，加快实施《桂林创新计划（2008—2010）》和科技“355工程”[14]。扶持企业申报专利、争创驰名商标和国家、自治区名牌，提高自主知识产权拥有量。大力推进以企业为主体、市场为导向、产学研相结合的区域技术创新体系建设。支持组建产业技术联盟，创建2家国家级、4家自治区级企业技术中心和工程技术研究中心。继续抓好生产力促进中心和科技企业孵化器建设，建立完善科技中介服务体系，推进科技成果转化。着力培养高层次科技创新人才，加强科学普及，实施民生科技行动，提高全民创新意识和科学素质。

提高开放合作水平。主动融入泛珠三角“9+2”、北部湾经济区、西江经济带等多区域合作。继续加大招商引资力度，创新招商方式，拓宽招商渠道，坚持大招商、招大商，加大“央企入桂”、“百企入桂”工作力度，健全招商引资目标管理责任制、评价考核机制。深化“大兑现，大服务”活动，进一步提高项目履约率、开竣工率、资金到位率。争取全年实际利用外资2.22亿美元以上，引进市外境内到位资金突破300亿元，引进亿元以上项目30个。继续实施“走出去”战略，引导企业开展境外投资。继续开展国际友好城市交流与合作，做好外事、侨务、港澳台工作。充分利用中国—东盟自由贸易区和中国—东盟博览会平台，加快桂林国家科技兴贸创新基地建设，优化出口产品结构，拓展东盟等新兴市场，完成外贸进出口总额7.80亿美元，增长11%。

（八）科学保护环境，提升生态桂林建设新水平

以建设生态山水名城为目标，以保护漓江为重点，加强生态建设，抓好节能减排，加快生态文明示范市创建步伐。

优化漓江流域生态。全面贯彻落实中央、自治区关于“科学保护漓江”重要批示精神，积极推动《漓江风景名胜区总体规划》和《广西壮族自治区桂林漓江生态环境保护条例》尽快出台。组织实施漓江源头水源林保护、两岸绿化美化、两岸及水域环境保护、两岸富民惠民、水域管理和景区旅游产品策划等6大工程。加强漓江水生资源保护和国家水产种质资源保护区建设，研究探索漓江流域生态补偿机制，争取列入国家生态补偿财政转移支付基金试点。

加强生态环境建设。组织实施《桂林生态市建设规划》，抓好《桂林市城市绿地系统规划》落实。全面开展造林绿化，实现人工造林34万亩，掀起新一轮植树造林高潮，创建“国家森林城市”。继续实施沼气池建设工程，新建沼气池1.25万座，可建池入户率超过78%。加强饮用水源地、水资源、生物多样性保护，推进会仙湿地、桂柳古运河恢复与保护，进一步抓好青狮潭水库等水环境污染治理。推进县乡土地利用规划和土地调查及成果应用工作，实施整村推进土地整治工程，切实保护耕地，节约集约用地。推进矿产资源整合开发，严厉打击乱采乱挖、无证开采、破坏矿产资源等违法行为。加强地质环境保护和地质灾害防治。全面推进生态市、生态县建设，积极创建全国环境优美乡镇，保持生态环境质量在全区的领先水平。

扎实抓好节能减排。严格落实节能减排工作责任制，继续推进环保专项行动，强化对污染源在线监控和监督性检测，促使污染源全面达标排放。抓好重点企业节能降耗，坚决淘汰落后产能，防止低水平重复建设，确保完成自治区下达的节能减排任务。完成各县和重点工业园区污水垃圾处理项目建设。提升医疗废弃物处置水平。积极推广应用节能节水技术和产品，推广绿色建筑，强化新建建筑、大型公共建筑节能。鼓励发展循环经济、低碳经济，积极争取列入“全国可再生能源建筑应用示范城市”，推进资源节约型、环境友好型社会建设。

　　（九）保障和改善民生，开创和谐桂林建设新局面

坚持以人为本，强化就业保障，发展社会事业，完善社会管理，解决好事关群众切身利益的突出问题，确保社会和谐稳定。

加强就业和社会保障。实施更加积极的就业政策，统筹推进城乡就业，稳定基本就业形势，全面实施首批国家级创业型城市创建工作，鼓励和支持下岗失业人员、大中专毕业生自主创业和自谋职业，扶持返乡农民工回乡创业，以全民创业促进就业。开发公益性岗位，加强市县乡三级职业技能培训，帮助更多城乡劳动力就业。实现新增农村劳动力转移就业8.34万人次以上。扎实推进劳动仲裁执法，创建和谐稳定劳动关系。以建立和完善覆盖城乡的社会保障体系为目标，抓好社会保险扩面征缴工作，实现企业职工基本养老保险参保35.85万人，失业保险参保24万人，基本医疗、工伤、生育保险参保人数分别达到95.5万、26.6万、24.8万。认真落实被征地农民养老保险和生活保障。发展社会福利事业和慈善事业，实施五保村建设，完善社会救助体系，确保受灾群众基本生活。

加快发展社会事业。全面推进素质教育，加强教师队伍建设，促进城乡义务教育均衡发展。深入推进职业教育攻坚工作，继续实施中职学生各项资助政策。积极支持驻桂林高等院校、市卫生学校、市交通技校新校区建设，做好临桂新区中小学布局规划。推进培智、聋哑、七中三校搬迁工程建设。实施国家基本公共卫生服务项目，逐步扩大医疗救助和医疗惠民范围。完成7所国家标准县级医院、18所乡镇卫生院建设，建立“15分钟”社区卫生服务圈。做好重大传染病防控，增强突发公共卫生事件的处置能力。大力发展全民健身事业，积极备战自治区第12届运动会。全面加强人口和计划生育工作，大力推进“诚信计生”工作，加快建立完善基层计生群众自治工作机制。促进民族事业发展，办好恭城瑶族自治县成立20周年庆典活动。组织力量科学编制“十二五”规划纲要及相关专项规划。做好全国第六次人口普查工作。

不断完善社会管理。健全基层自治机制，巩固和扩大基层民主。建立和完善政府依法行政各项制度，积极创建法治城市、法治县区，大力开展“和谐建设在基层活动”，争创全国普法依法治理先进城市。以公安信息化建设为抓手，完善社会治安防控体系，扎实抓好社会治安综合治理工作。健全突发公共事件应急机制、社会矛盾调处机制和信访工作机制，加大社会矛盾纠纷排查化解力度，推动人民调解、行政调解、司法调解相衔接，提高突发性群体性事件处置能力。强化安全生产监督管理，有效防范和坚决遏制重特大安全事故发生。做好源头监控和市场监管，确保食品药品安全。加强国防动员和民兵预备役建设，不断完善国防基础设施建设，扎实做好双拥工作，争创全国军民共建双拥模范城市。

深入实施惠民工程。继续从解决群众最关心、最直接、最紧迫的问题入手，筹集资金10亿元以上，全力办好10项惠民工程：

1.教育惠民工程。①实施中小学校舍安全工程。完成中小学（含中职）校舍加固改造、重建项目任务不少于50个，改善中小学校舍安全状况。②实施库区普通高中学生免除学费工程。对进入中等职业学校的库区移民给予学费资助。

2.医疗卫生保障惠民工程。①实施基本公共卫生保障工程。建立居民健康档案、健康教育、预防接种、传染病防治等九类基本公共卫生服务项目[15]，使城乡居民逐步享有均等化的基本公共卫生服务。②实施母婴健康“一免二补”幸福工程。对新婚夫妇实行免费婚前医学检查，对孕妇产前筛查及新生儿疾病筛查实行补助。③建立困难危重孕产妇救助基金。④实施“健康快车”复明工程。完成全市白内障患者复明手术1000例以上。

3.社会保障惠民工程。①继续完善城镇居民基本医疗保险制度。实现参保率达90%以上，补助标准从每人100元提高到120元。②继续完善新型农村合作医疗制度，补助标准从每人80元提高到120元，力争参合率达95%以上。③实施关闭破产企业退休人员医疗保障工程。为关闭破产国有困难企业退休人员、国有困难企业职工办理城镇基本医疗保险。④实施“阳光家园计划”建设工程。建设10个残疾人日间托养机构，为2000名智力、精神和重度残疾人提供日间照料和居家托养服务。

4.文化惠农工程。①实施村级公共服务中心示范工程。在50个村实现一个灯光球场、一个文艺舞台、一栋综合楼（活动室），组建一支农民文艺队、一支农民篮球队。②继续实施20户以上村村通广播电视工程。

5.安居惠民工程。①新开工建设2万平方米廉租房。②继续落实少数民族聚居村寨防火工程。对50户以上连片木结构民族村寨实施防火综合整治，解决部分民族村寨防火问题。

6.农村基础设施建设工程。①完成55个自然村（屯）新农村建设试点工作。②实施村（屯）道路建设。完成20个行政村通油路或水泥路项目，修（扩）建250条贫困村通村（屯）道路，继续实施1500个自然村（屯）内道路硬化。③实施农村饮水安全工程360处，解决20万农村人口饮水安全问题，其中在全市贫困村新建人畜饮水工程50处，解决贫困村人畜饮水问题。

7.城乡社会服务工程。①新建、改扩建计生服务站、所20个，村级健康家庭服务室100个。②继续实施“万村千乡”市场工程。新建和改造农家店280家。

8.“放心菜”工程。实施农贸市场蔬菜质量安全检测室建设工程，在市区铁路、七星、东莲、金鸡岭和中北5个农贸市场及12县城农贸市场各建1个蔬菜质量安全检测室。

9.城市水环境综合整治工程。实施“两江四湖”二期——桃花江、南溪河、小东江综合治理工程，改善老城生态景观环境，增加市民休闲场所。

10.城市交通环境综合治理工程。继续实施市区重要交通节点改造，重点建设福利路、芳香路、阳江南路，改善市区交通条件。实施市区部分街区路灯安全隐患治理工程，消除安全隐患。

各位代表，新的形势和任务给政府工作提出了新的更高要求，我们必须加强自身建设，提高领导科学发展和驾驭复杂局面的能力，建设为民、务实、廉洁、高效政府。一是切实转变职能。坚持有序推进政府机构改革，健全和执行重大决策的规则和程序，切实做到依法决策、科学决策、民主决策。二是切实转变作风。坚持迎难而上的精神状态和雷厉风行的工作劲头，严格实行首问负责制、限时办结制和责任追究制，戒骄戒懒戒空戒虚戒假戒奢，把狠抓落实作为政府工作的第一要求，用实干体现能力，用落实体现水平，提高政府行政效能。三是切实执政为民。坚持问政于民、问需于民、问计于民，关心群众疾苦，倾听群众呼声，使政府的发展思路、工作部署、政策措施更加符合人民群众的愿望。四是切实加强督查。坚持联合督查、专项督查、全程跟踪督查，及时掌握和协调解决推进重点工作中出现的困难和问题，确保各项工作落到实处。五是切实廉洁从政。坚持从严治政，完善惩治和预防腐败机制，加大对重点领域的监管，全面落实自治区关于建设“优质工程、安全工程、阳光工程、廉政工程”要求，确保办成事，不出事。六是切实依法行政。坚持自觉接受市人大及其常委会的监督，重视市政协的民主监督，认真听取各民主党派、工商联、人民团体和社会各界人士的意见，集思广益，形成合力，共同完成全年目标任务。

各位代表！新的一年，我们又站在了新的历史起点上。跋山涉水，自当勇攀高峰；任重道远，更需快马加鞭。让我们紧密地团结在以胡锦涛同志为总书记的党中央周围，以邓小平理论和“三个代表”重要思想为指导，深入学习实践科学发展观，在市委的正确领导下，团结和调动一切积极力量，以昂扬向上、积极进取的工作状态，以只争朝夕、奋发有为的工作干劲，以脚踏实地、优质高效的工作作风，齐心协力，开拓创新，顽强拼搏，为继续保持经济平稳较快增长，加快建设现代化国际旅游名城、历史文化名城、生态山水名城而努力奋斗！
